# Supplementary material for: Application of Environmental DNA for Assessing the Distribution and Biomass of Brachymystax lenok Tsinlingensis in the Zhouzhi Heihe River
Source: Animals (Basel). 2025 Mar 28;15(7):977. doi: 10.3390/ani15070977 (PMC11987999; doi:10.3390/ani15070977)
Supplement: Supplementary file 1 [file animals-15-00977-s001.zip › animals-3479957-supplementary.pdf]

Preparation of plasmid and determination of the threshold in qPCR

Two fin samples of *B. lenok tsinlingensis* were obtained from Yili Culture Co., Ltd (Taibai County; 33°02′N and 107°37′E), gathered, and stored in 95% ethanol until DNA extraction. We used the TIANamp Genomic DNA Kit (TIANGEN, Beijing) to extract the total genomic DNA from the fin tissue samples and PCR products of the target sequences using a primer pairs from Li et al., (2017) were inserted into the pMD 19-T Vector (TaKaRa, Dalian, China) [45]. The plasmid DNA samples were progressively diluted, with dilution ranging from 10^{-1} to 10^{-11} ng/μL and eight replicates were performed for each dilution concentration during the standard curve quantification process. The average r^2 for curves used in this study was 0.98. This approach was selected to define the limit of detection (LOD; the minimum amount of target DNA sequence that can be detected in the sample) and the limit of quantification (LOQ; the lowest amount of target DNA that yields an acceptable level of precision and accuracy) for *B. lenok tsinlingensis* DNA [46].
